# Temperature‐Dependent Surface Enrichment Effects in Binary Mixtures of Fluorinated and Non‐Fluorinated Ionic Liquids

**DOI:** 10.1002/chem.201904438

**Published:** 2020-01-07

**Authors:** Bettina S. J. Heller, Matthias Lexow, Francesco Greco, Sunghwan Shin, Gabriel Partl, Florian Maier, Hans‐Peter Steinrück

**Affiliations:** ^1^ Lehrstuhl für Physikalische Chemie II Friedrich-Alexander-Universität Erlangen-Nürnberg Egerlandstraße 3 91058 Erlangen Germany; ^2^ Institut für Allgemeine Anorganische und Theoretische Chemie Leopold-Franzens-Universität Innsbruck Innrain 80–82 6020 Innsbruck Austria

**Keywords:** ionic liquids, mixtures, photoelectron spectroscopy, surface enrichment

## Abstract

Using angle‐resolved X‐ray photoelectron spectroscopy (ARXPS), we investigate the topmost nanometers of various binary ionic liquid (IL) mixtures at different temperatures in the liquid state. The mixtures consist of ILs with the same [PF_6_]^−^ anion but two different cations, namely 3‐methyl‐1‐(3,3,4,4,4‐pentafluorobutyl)imidazolium hexafluorophosphate, [PFBMIm][PF_6_], and 1‐butyl‐3‐methylimidazolium hexafluorophosphate, [C_4_C_1_Im][PF_6_], with 10, 25, 50 and 75 mol % content of [PFBMIm][PF_6_]. We observe a preferential enrichment of the fluorinated chain in the topmost layer, relative to the bulk composition, which is most pronounced for the lowest content of [PFBMIm][PF_6_]. Upon cooling the mixtures stepwise from 95 °C until surface charging effects in XPS indicate solidification, we observe a pronounced increase in surface enrichment of the fluorinated chain with decreasing temperature in the liquid state. In contrast to the mixtures with lower [PFBMIm][PF_6_] contents, cooling the 75 mol % mixture additionally shows an abrupt decrease of the fluorinated chain signal before complete solidification occurs, which is assigned to partial precipitation effects.

## Graphical abstract

**Mixtures of the ionic liquids** [PFBMIm]‐[PF_6_] and [C_4_C_1_Im][PF_6_] exhibit a very pronounced surface enrichment of the fluorinated chain. This effect is most pronounced at low contents of [PFBMIm][PF_6_], and increases with decreasing temperature.

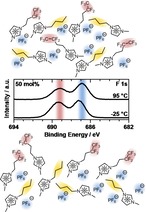

## Introduction

Ionic liquids (ILs) carrying fluorinated alkyl chains, in the following called fluorinated ILs, represent an interesting class of ILs because they often exhibit unique properties such as high thermal and chemical stability, and high gas solubility. Moreover, they commonly show low surface tension and/or are chemically and biologically inert.^1^ Therefore, they are potential candidates to substitute fluorinated organic compounds in different applications, for example, as surfactants, gas absorbents, lubricants and refrigerants.1a, 2 It has also been shown that fluorinated ILs can be used as gas carriers,1a, 1d electrolytes in fuel and solar cells, and in lithium batteries,1b, 2, 3 in catalysis^2^, ^4^ and in many more applications.

While non‐fluorinated ILs are known to form bulk nanostructures consisting of polar and nonpolar domains, fluorinated ILs typically exhibit additional nonpolar fluorous domains, in which the fluorinated chains preferentially agglomerate.1a–1c, 5 Alkylated and fluorinated chains can be present either in one single IL (e.g. alkyl chains in the cation and fluorinated chains in the anion or vice versa1b), or in IL mixtures with one IL containing alkyl chains and the other one containing fluorinated chains. Changing the relative chain length in one IL or the molar ratio of the two ILs in the mixture influences the related properties and the domain structure.^5^, ^6^ It should be emphasized that using IL mixtures allows for fine‐tuning the properties in a very subtle way. Applying IL mixtures instead of using one neat IL becomes even more beneficial in the context of the European chemical registration process REACH: If one could achieve specific properties by employing adequate mixtures of pre‐registered ILs instead of synthesizing a new task‐specific IL, a lengthy and costly registration process could be avoided. The enormous effort to register a new chemical is demonstrated by the fact that among the many ILs synthesized today, as of June 2019 only eight ILs based on the standard 1‐ethyl‐3‐methylimidazolium cation (see Table S1 in the Supporting Information) have been approved by REACH and only about six further imidazolium salts with melting points below 100 °C have been registered.^7^

Many investigations have been carried out in the last decades on IL mixtures and their composition‐dependent physico‐chemical bulk properties such as viscosity, thermal behavior, density and molar volume, conductivity, solvation abilities, influence on chemical reactivity as well as bulk microscopic structure (e.g., see reviews in Ref. 8 and references therein, and Ref. 9). In contrast, much less studies are available on the surface properties of IL mixtures, despite the fact that the composition of the topmost layers can differ considerably from the bulk composition.^6^, 8b, 10 In many of the aforementioned applications, particularly those involving systems where a thin IL film coats a high surface area support, the surface, that is, the IL/vacuum(gas) interface plays an important role for the overall performance. Therefore, investigations of the topmost layers of IL mixtures are getting more into the focus of research. A variety of studies has been performed, using reactive‐atom scattering with laser‐induced fluorescence detection (RAS‐LIF),6a, 6c neutron scattering,6c small‐angle X‐ray scattering6c and X‐ray reflectivity,^11^ time of flight secondary ion mass spectrometry (TOF‐SIMS),^12^ Rutherford backscattering spectroscopy (RBS),12a, 13 low‐energy ion scattering (LEIS),^14^ X‐ray photoelectron spectroscopy (XPS)6b, 10a, 15 and molecular dynamics (MD) simulations.1b, 5, 6, 13b, 16

Surface studies of ILs with both alkylated and fluorinated chains are quite rare. In case of neat ILs, Luís et al.1b recently measured the surface tension of [C_*n*_C_1_Im][C_4_F_9_SO_3_] ([C_4_F_9_SO_3_]=perfluorobutanesulfonate) with alkyl chain lengths *n=*2, 4, 6, 8, 10 and 12, and correlated the derived values with MD simulations. The latter revealed that for cations with less than four carbon atoms in the alkyl chain, the outer surface is dominated by the presence of the fluorinated butyl chains of the anion, with the polar head groups preferentially forming a confined sub‐surface layer. Longer alkyl chains (*n*>4) start to penetrate the fluorinated surface layer pushing the polar head groups of the anion and cation even further away from the outer surface. These findings are in line with the decrease in measured surface tension of the ILs up to *n=*8, where a maximum number of both fluorinated and alkylated chains (and a minimum number of polar head groups) are present at the outer surface. For even longer alkyl chains (*n*>8), the surface tension increases again due to the fact that the longer alkyl chains dominate the outer surface, as evidenced by MD simulations.

In the case of [C_8_C_1_Im]_1−*x*_[C_8_C_1_ImF_13_]_*x*_[Tf_2_N] mixtures, RAS‐LIF measurements performed by Smoll et al.6a showed that the fluorinated chain is again preferentially enriched at the surface. By investigating different stoichiometries, they found a large surface excess of the fluorinated chains compared to the nominal bulk composition, particularly at the lowest mole fraction of the fluorinated IL.

**Figure 1:**
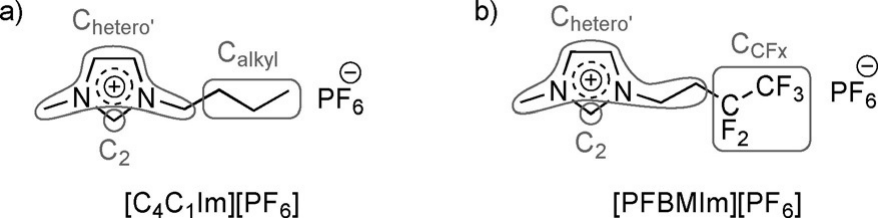
Molecular structures of a) 1‐butyl‐3‐methylimidazolium hexafluorophosphate, [C_4_C_1_Im][PF_6_], and b) 3‐methyl‐1‐(3,3,4,4,4‐pentafluorobutyl)imidazolium hexafluorophosphate, [PFBMIm][PF_6_], including the denotation of the carbon atoms, C_2_, C_hetero′_, C_alkyl_ and C_CFx_, in gray.

In this study, we present a detailed angle‐resolved XPS (ARXPS) study under clean ultra‐high vacuum (UHV) conditions on neat [PFBMIm][PF_6_] and [C_4_C_1_Im][PF_6_] (see Figure 1), and mixtures thereof in four molar ratios (10, 25, 50 and 75 mol %). Both ILs are comprised of the same hexafluorophosphate ([PF_6_]^−^) anion and similar imidazolium‐based cations. In [C_4_C_1_Im]^+^, the butyl chain is fully hydrogenated, and in [PFBMIm]^+^ its terminal ethyl moiety is fluorinated. We analyzed the temperature dependence of the surface composition upon cooling from 95 °C, where all mixtures are in their liquid state, until their solidification. We observe a very pronounced surface enrichment of the fluorinated [PFBMIm]^+^ chains relative to the bulk composition, which increases with decreasing temperature and decreasing mole fraction of [PFBMIm][PF_6_].

## Experimental Section

Materials: [C_4_C_1_Im][PF_6_] was purchased from Iolitec (purity 99.5 %). The synthesis of [PFBMIm][PF_6_] has been reported before.^17^ All neat ILs investigated in this study were used as supplied. To prepare the IL mixtures, acetonitrile (Sigma–Aldrich, purity 99.8 %) was used as a co‐solvent to ensure proper mixing of the ILs. For the ARXPS measurements of the neat ILs and the mixtures, a layer of up to 0.5 mm thickness was prepared on a molybdenum sample holder reservoir. Subsequently, the sample holder was placed into the load‐lock of our vacuum chamber and degassed for at least twelve hours.

Angle‐resolved X‐ray photoelectron spectroscopy (ARXPS): The ARXPS experiments were performed in our DASSA (Dual Analyzer System for Surface Analysis) setup, for details see Ref. 18. Simultaneous acquisition of ARXP spectra at two fixed emission angles of *ϑ*=0° (normal emission) and 80° (grazing emission) with respect to the surface normal of a horizontally mounted sample was achieved by two hemispherical energy analyzers (ARGUS‐type). This reduces the total measurement time and thus the exposure of the sample to X‐rays by a factor of two, which in turn minimizes radiation damage. Furthermore, the spectra at 0° and 80° correspond to the sample under exactly identical conditions. A monochromated X‐ray source with Al K_α_ radiation (XM 1000, *hν*=1486.6 eV, 238 W) was used as X‐ray source. Survey scans were recorded with a pass energy of 150 eV and region scans with 35 eV; for the latter, the overall energy resolution is 0.4 eV. The binding energy scale was referenced to the Fermi level of Au.

In organic matter, the information depth of photoelectrons after excitation with Al K_α_ radiation at 0° is 7 to 9 nm (depending on the kinetic energy). At 80°, it decreases to 1.0 to 1.5 nm, making the measurement very surface sensitive: ≈80 % of the signal originates from the topmost molecular layer. Each set of 80° spectra was scaled up by an individual geometry factor to compensate for lower intensity compared to 0° spectra.^18^ After this normalization, intensity differences between 80° and 0° emission angle directly reflect a higher/lower concentration of the respective species at the surface than in the bulk. This allows us to reveal surface enrichment and molecular orientation effects.

For the quantitative analysis of the spectra, we used atomic sensitivity factors (ASFs).^18^ As the C 1s signal from the CF_3_ group of the [PFBMIm]^+^ cation overlaps with the shake‐up of the aromatic system of the cation, the intensity of this peak is set equal to that of the CF_2_ peak. CasaXPS (version 2.3.16) was used for subtracting the background and for peak fitting (pseudo‐Voigt function with 30 % Lorentzian contribution). In the F 1s, N 1s and P 2p spectra, a two‐point linear background was subtracted, whereas a three‐point linear background was used for the C 1s spectra. The P 2p signal is composed of the spin‐orbit‐split 2p_1/2_ and 2p_3/2_ components, which have the same full width at half maximum (FWHM), are separated by 0.9 eV, and have an area ratio of 1:2. The FWHM of the F 1s peaks of the CF_x_ groups (*x=*2 and 3) and the [PF_6_]^−^ anions are 1.94±0.1 and 1.47±0.1 eV, respectively. For the C 1s peaks (see Figure 1 for nomenclature), the following constraints were applied: For neat [PFBMIm][PF_6_] and for the mixtures, C_hetero’_ is 1.33 times wider than C_2_ and these two peaks are separated by 1.02 eV; for neat [C_4_C_1_Im][PF_6_], C_2_ and C_hetero’_ are separated by 0.9 eV, and the FWHM of the C_hetero’_ and C_alkyl_ peaks is set to 1.1 and 1.11 times that of C_2_, respectively.

The sample temperature was measured with a type K thermocouple attached to the molybdenum sample reservoir with an accuracy of ±5 °C, and a stability of ±1 °C.^19^

## Results and Discussion

We investigated various mixtures of 3‐methyl‐1‐(3,3,4,4,4‐pentafluorobutyl)imidazolium hexafluorophosphate, [PFBMIm][PF_6_], and 1‐butyl‐3‐methylimidazolium hexafluorophosphate, [C_4_C_1_Im][PF_6_], (see Figure 1 for structures) by temperature‐dependent ARXPS. The two ILs have different cations but the same anion, [PF_6_]^−^. Apart from the neat ILs, the mixtures with molar ratios of 10, 25, 50 and 75 mol % of [PFBMIm][PF_6_] were studied. In the following, we first present the data for the two neat ILs and the mixtures at 95 °C. At this temperature, all ILs and mixtures are liquid (the glass transition temperature of [C_4_C_1_Im][PF_6_] is −77 °C^20^ and the melting point of [PFBMIm][PF_6_] is 66 °C^17^). We restricted our investigations to 95 °C and below because of a rise in background pressure to above 5×10^−9^ mbar at higher temperatures. Thereafter, we discuss their temperature‐dependent behavior. In all figures, the spectra in black correspond to an emission angle of 0°, and the red spectra to 80°.

### Neat [C_4_C_1_Im][PF_6_]

**Figure 2:**
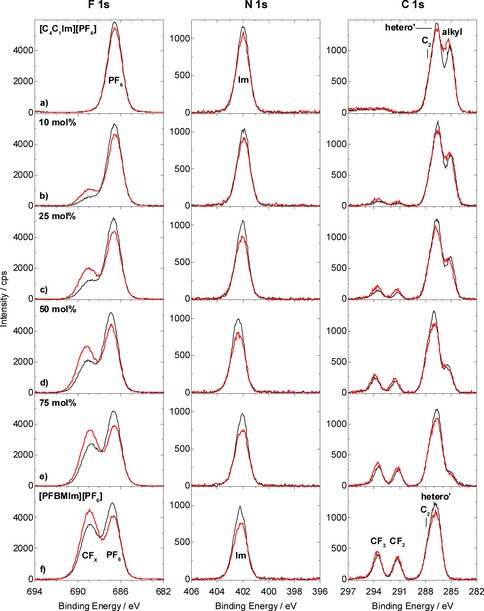
F 1s (left), N 1s (center) and C 1s (right) spectra, at 0° (black) and 80° (red) emission: a) Neat [C_4_C_1_Im][PF_6_], b)–e) mixtures of [PFBMIm][PF_6_] with [C_4_C_1_Im][PF_6_] at molar ratios of b) 10 mol % [PFBMIm][PF_6_], c) 25 mol % [PFBMIm][PF_6_], d) 50 mol % [PFBMIm][PF_6_] and e) 75 mol % [PFBMIm][PF_6_], and f) neat [PFBMIm][PF_6_]. All spectra were acquired at a sample temperature of 95 °C.

In Figure 2 a, the 0° emission XP spectra of [C_4_C_1_Im][PF_6_] are depicted, for a temperature of 95 °C. The F 1s spectrum (left panel) shows the F_PF6_ peak of the [PF_6_]^−^ anion at 686.6 eV. The single peak in the N 1s region (center panel) at 402.0 eV is assigned to the imidazolium nitrogen atoms, N_Im_. In the C 1s spectrum (right panel), the C_alkyl_ peak at lowest binding energy of ≈285 eV stems from the three alkyl carbon atoms only bound to hydrogen and carbon atoms, and the peak at higher binding energy consists of two contributions: The smaller C_2_ peak at 287.5 eV is due to the carbon atom bound to two nitrogen atoms, and the C_hetero’_ peak at 286.6 eV due to carbon atoms bound to one nitrogen atom. In the P 2p region (see Figure S1a in the Supporting Information), the unresolved spin‐orbit peaks of the phosphorus atom of the [PF_6_]^−^ anion are centered at 136.9 eV. Notably, no signals of possible surface‐active contaminations^21^ from the synthesis are observed in the O 1s and Si 2p region (see Figure S1a in the Supporting Information), confirming the purity of [C_4_C_1_Im][PF_6_]. Within the margin of error (±10 %), the quantitative analysis at 0° agrees very well with the nominal composition of [C_4_C_1_Im][PF_6_] (see Table 1 a).

**Table 1:** Quantitative analysis of the 0° and 80° XP spectra at 95 °C. Nominal and experimentally determined contents (mole fraction) are given for all elements using ASFs from Ref. 18. a) Neat [C_4_C_1_Im][PF_6_], b)–e) mixtures of [PFBMIm][PF_6_] with [C_4_C_1_Im][PF_6_] at molar ratios of b) 10 mol % [PFBMIm][PF_6_], c) 25 mol % [PFBMIm][PF_6_], d) 50 mol % [PFBMIm][PF_6_] and e) 75 mol % [PFBMIm][PF_6_], and f) neat [PFBMIm][PF_6_].

Core levels	F 1s	F 1s	N 1s	C 1s	C 1s	C 1s	C 1s	P 2p
a) [C_4_C_1_Im][PF_6_]	F_CFx_	F_PF6_	N_Im_	C_CFx_	C_2_	C_hetero‘_	C_alkyl_	P_PF6_
BE/eV	–/–	686.6	402.0	–/–	287.5	286.6	285.1	136.9
ASF	1.00	1.00	0.46	0.30	0.30	0.30	0.30	0.46
*nominal*	*–/–*	*6.0*	*2.0*	*–/–*	*1.0*	*4.0*	*3.0*	*1.0*
0° emission	–/–	6.4	2.0	–/–	0.9	3.8	2.9	1.1
80° emission	–/–	6.0	1.9	–/–	0.9	3.7	3.4	1.1
								
b) 10 mol % [PFBMIm][PF_6_]	F_CFx_	F_PF6_	N_Im_	C_CFx_	C_2_	C_hetero‘_	C_alkyl_	P_PF6_
*nominal*	*0.5*	*6.0*	*2.0*	*0.2*	*1.0*	*4.1*	*2.7*	*1.0*
0° emission	0.9	6.2	2.0	0.2	0.7	4.3	2.3	1.1
80° emission	1.6	5.4	1.8	0.4	0.6	4.3	2.3	1.1
								
c) 25 mol % [PFBMIm][PF_6_]	F_CFx_	F_PF6_	N_Im_	C_CFx_	C_2_	C_hetero‘_	C_alkyl_	P_PF6_
*nominal*	*1.3*	*6.0*	*2.0*	*0.5*	*1.0*	*4.3*	*2.3*	*1.0*
0° emission	1.8	6.1	1.9	0.5	0.8	4.4	1.8	1.1
80° emission	3.0	5.2	1.7	0.8	0.7	4.2	1.7	1.0
								
d) 50 mol % [PFBMIm][PF_6_]	F_CFx_	F_PF6_	N_Im_	C_CFx_	C_2_	C_hetero‘_	C_alkyl_	P_PF6_
*nominal*	*2.5*	*6.0*	*2.0*	*1.0*	*1.0*	*4.5*	*1.5*	*1.0*
0° emission	3.1	6.1	1.9	0.9	0.9	4.4	1.2	1.1
80° emission	4.5	5.1	1.7	1.2	0.8	4.1	1.0	1.0
								
e) 75 mol % [PFBMIm][PF_6_]	F_CFx_	F_PF6_	N_Im_	C_CFx_	C_2_	C_hetero‘_	C_alkyl_	P_PF6_
*nominal*	*3.8*	*6.0*	*2.0*	*1.5*	*1.0*	*4.8*	*0.8*	*1.0*
0° emission	4.2	6.0	1.9	1.4	1.0	4.6	0.5	1.1
80° emission	5.8	4.9	1.7	1.8	1.0	4.2	0.4	1.0
								
f) [PFBMIm][PF_6_]	F_CFx_	F_PF6_	N_Im_	C_CFx_	C_2_	C_hetero‘_	C_alkyl_	P_PF6_
BE/eV	688.9	686.8	402.2	291.3/293.6	287.8	286.8	–/–	137.0
*nominal*	*5.0*	*6.0*	*2.0*	*2.0*	*1.0*	*5.0*	*–/–*	*1.0*
0° emission	5.6	6.0	1.9	1.8	1.0	4.6	–/–	1.1
80° emission	7.0	5.0	1.7	2.1	1.0	4.1	–/–	1.0

When comparing the 80° (red) and 0° (black) emission spectra of [C_4_C_1_Im][PF_6_] in Figure 2 a, we find a slight increase of the C_alkyl_ signal at 80°, indicating a slight enrichment of the butyl chain at the IL/vacuum interface. Such an enrichment is generally known in literature for non‐functionalized [C_*n*_C_1_Im]^+^ cations with alkyl chains with *n*≥4.^22^ In line with this enrichment, we find a decrease of the F_PF6_ and N_Im_ signals at 80°, indicating a slight depletion of the [PF_6_]^−^ anion and the imidazolium ring of the cation from the IL/vacuum interface.

### Neat [PFBMIm][PF_6_]

The XP spectra of [PFBMIm][PF_6_] are depicted in Figure 2 f. We recently investigated ultrathin layers of this IL on a Ag(111) surface in the monolayer range, using a non‐monochromated Al K_α_ X‐ray source in another XP setup.^17^ In the following, we now present the high‐resolution spectra of a thick film.

At 0° emission (black spectrum), the F 1s spectrum (left panel) displays two peaks, which arise from the fluorine atoms in two different chemical environments: The peak at 688.9 eV stems from the five F_CFx_ atoms (CF_x_=CF_2_ and CF_3_ groups) of the fluorinated chain in the [PFBMIm]^+^ cation, and the peak at 686.8 eV from the six F_PF6_ atoms of the [PF_6_]^−^ anion. In the N 1s region (center panel), a single peak is observed at 402.2 eV, due to the N_Im_ atoms of the imidazolium ring. In the C 1s region (right panel), the peaks at 293.6 and 291.3 eV stem from the C_CF3_ and C_CF2_ groups of the fluorinated butyl chain of the [PFBMIm]^+^ cation. The peak at lower binding energy consists of two peaks due to the C_2_ atom at 287.8 eV and the C_hetero’_ atoms at 286.8 eV. The P 2p spectrum (Figure S1f in the Supporting Information) displays the spin‐orbit‐split P_PF6_ peak of the anion centered at 137.0 eV. Again, the absence of O 1s and Si 2p signals (see Figure S1f in the Supporting Information) indicates that [PFBMIm][PF_6_] is clean.^21^ This is further confirmed by the fact that the quantitative analysis at 0° agrees very well with the nominally expected composition of [PFBMIm][PF_6_] (see Table 1 f).

Next, we compare the spectra at 80° in Figure 2 f to those at 0° emission. In the F 1s spectrum, clearly a strong increase of the F_CFx_ peak of the fluorinated chain at 80° is observed that goes along with a pronounced decrease of the F_PF6_ signal. This behavior indicates that the fluorinated chain of the cation is enriched at the IL/vacuum interface, while the [PF_6_]^−^ anion is surface‐depleted. The enrichment of the fluorinated chain is also reflected by the increase of the C_CF3_ and C_CF2_ peaks in the C 1s region at 80°. This increase goes along with a decrease of the C_2_ and C_hetero’_ peaks and also of the N_Im_ signal in the N 1s region; both observations indicate a surface depletion of the imidazolium ring of the [PFBMIm]^+^ cation. In the P 2p spectrum, changing the emission angle from 0° to 80° emission leads to a small decrease.

### Mixtures of [PFBMIm][PF_6_] and [C_4_C_1_Im][PF_6_]

To search for preferential enrichment effects of different cations in mixtures of [PFBMIm][PF_6_] and [C_4_C_1_Im][PF_6_] as a function of the composition, we studied four mixtures with molar ratios of 10, 25, 50 and 75 mol % of [PFBMIm][PF_6_]. The corresponding XP spectra are depicted in Figure 2 b–2e, respectively, for emission angles of 0° (black) and 80° (red).

We start with the discussion of the F 1s spectra (left panel) at 0°. In addition to the F_PF6_ peak of the anion at 686.7 eV, all spectra display the F_CFx_ peak of the cation at 688.9 eV. The intensity of the latter peak increases with increasing molar ratio, as is expected. In all cases, the N 1s spectra (center panel) show a single peak, N_Im_, at 402.1 eV originating from the nitrogen atoms in the imidazolium rings of both the [PFBMIm]^+^ and the [C_4_C_1_Im]^+^ cations. In the C 1s region (right panel), we find five peaks for all mixtures. The peaks at 293.6 and 291.3 eV are due to the C_CF3_ and C_CF2_ atoms of the [PFBMIm]^+^ cation, respectively. The C_alkyl_ peak of the [C_4_C_1_Im]^+^ cation is observed at 285.1 eV, and the C_hetero’_ peak with the C_2_ shoulder is found at 286.7 and 287.7 eV, respectively. The spin‐orbit‐split P 2p peaks of the [PF_6_]^−^ anion are centered at 137.0 eV (see Figure S1b–S1e in the Supporting Information). Within the margin of error (±0.2 eV) the peaks in all regions have the same binding energy like in the neat ILs. Again, no signals are detected in the Si 2p and O 1s spectra (Figure S1b–S1e in the Supporting Information), verifying that no contaminations are transferred to the mixture by using acetonitrile as a co‐solvent when preparing the mixtures. Within the margin of error, the quantitative analyses are in line with the nominal compositions of the mixtures of [PFBMIm][PF_6_] and [C_4_C_1_Im][PF_6_], except for a general too high content of F_CFx_ atoms and a too low content of C_alkyl_ atoms, derived from the bulk‐sensitive measurements at 0° (Table 1 b–1f). This observation indicates that strong enrichment and depletion effects are not only visible in 80° measurements but also in 0° emission (see also below). Recently, the deviation from the nominal bulk values at 0° emission, and therefore the pronounced enrichment/depletion of one of the species of mixtures, was reported by our group and others.6b, 10a

**Figure 3:**
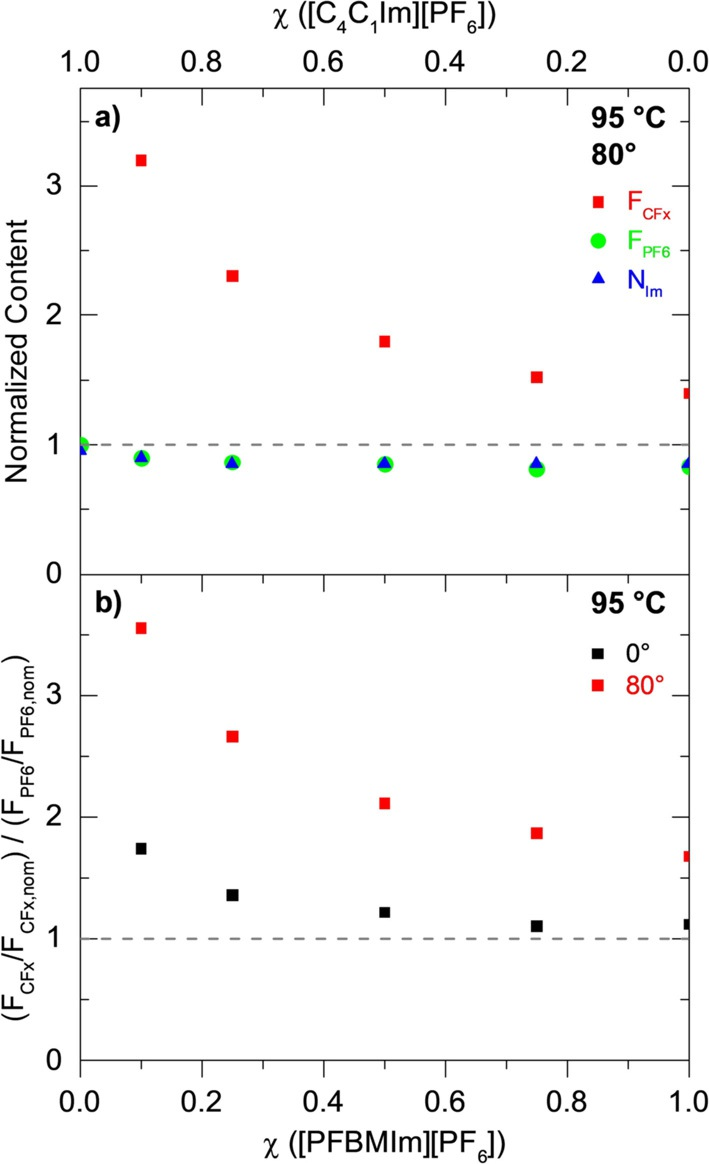
a) Normalized content of F_CFx_ (red squares), F_PF6_ (green circles) and N_Im_ (blue triangles) for 80° emission (data from Figure 2 and Table 1 a–1f). In each case, the experimentally determined content is divided by the nominal content. b) Ratio of the normalized F_CFx_ and F_PF6_ contents, at 0° (black) and 80° (red) emission angle. In all cases, the sample temperature was 95 °C. The dashed horizontal lines indicate the nominal compositions.

Next, we analyze the XP spectra at 80°. For all mixtures, a significant increase of the F_CFx_ peak at 688.9 eV indicates a clear surface enrichment of the fluorinated chain, similar to the observation for neat [PFBMIm][PF_6_]. Interestingly, the increase of the F_CFx_ signal at 80° relative to that at 0° is most pronounced for the mixture with the lowest molar ratio of 10 mol % [PFBMIm][PF_6_]. With increasing molar ratio, this enhancement continuously decreases. To visualize this behavior, we plotted the normalized F_CFx_ content, that is, the experimentally determined content (mole fraction) divided by the nominal content (see Table 1) as a function of the molar ratio in Figure 3 a (red squares). A value of 1.0 would represent the situation, where the surface composition is identical to that in the bulk, that is, no surface enrichment. Figure 3 a shows a strong increase of the normalized F_CFx_ content with decreasing molar ratio of [PFBMIm][PF_6_]. This behavior clearly indicates that the surface enrichment of the fluorinated chain in the outermost layer (relative to the bulk) strongly increases for low molar ratios, that is, the surface of the mixture is preferentially terminated with the fluorinated chain.

This enrichment of the fluorinated chain is also detected in the C 1s region (Figure 2 b–2e, right panel) at 80°, where we observe an intensity increase of the C_CF3_ and C_CF2_ peaks. Generally, the effect is less pronounced than in the F 1s region, which is due to the higher kinetic energy of the C 1s photoelectrons of around 1200 eV as compared to around 800 eV for F 1s, which leads to a larger information depth of the former. In contrast to the increase of the CF_x_ signal of the fluorinated chains of [PFBMIm]^+^, the C_alkyl_ signal of the alkyl chains of [C_4_C_1_Im]^+^ has decreased at 80° for all IL mixtures. This behavior is in contrast to the situation for neat [C_4_C_1_Im][PF_6_] in Figure 2 a and for neat ILs with longer alkyl chains,^22^ and indicates that the alkyl chains in the mixtures studied here are depleted from the liquid/vacuum interface. The driving force is a competing effect between the fluorinated chain of the [PFBMIm]^+^ cation and the non‐fluorinated side chain of the [C_4_C_1_Im]^+^ cation: The selective enrichment of the [PFBMIm]^+^ cations is attributed to a (typically) larger surface tension of ILs with an alkyl chain compared to a fluorinated chain.1b, 6a, 23

The analysis of the F_PF6_ and P_PF6_ signals of the [PF_6_]^−^ anion, as well as the C_hetero’_ and N_Im_ signals of the imidazolium ring of the cation at 80° show for all mixtures (Figure 2 b–2e and Figure S1b–S1e in the Supporting Information) more or less the same behavior as for the two neat ILs (Figure 2 a and 2f). The 80° signals are up to 20 % smaller than the 0° signals, which results from the damping of the corresponding signals by the surface‐enriched alkyl and fluorinated chains. In Figure 3 a, we plotted the normalized F_PF6_ and N_Im_ contents (green circles and blue triangles, respectively) as a function of the molar ratio. For both, we find values smaller than 1.0, which reflect the described damping. The data corresponding to the anion and the imidazolium ring are identical, indicating that both are at the same distance from the surface.

To visualize the relative enrichment of the fluorinated chain of the cation relative to the anion (and thus also to the cation head group), we plot the ratio of the normalized F_CFx_ and F_PF6_ contents (data from Figure 3 a), that is, (F_CFx_/F_CFx,nom_) / (F_PF6_/F_PF6,nom_), as a function of the molar ratio in Figure 3 b. We will use this type of presentation later for the temperature‐dependent studies. The strong increase of the 80° signal with decreasing molar ratio again reflects the pronounced surface enrichment of the fluorinated chain of the [PFBMIm]^+^ cation. Interestingly, we also find a clear increase of the ratio of the normalized F_CFx_ and F_PF6_ contents for 0°. This behavior again indicates that strong selective enrichment effects are not only observed at 80° but also at 0° (see above).

### Temperature dependence of surface enrichment

**Figure 4:**
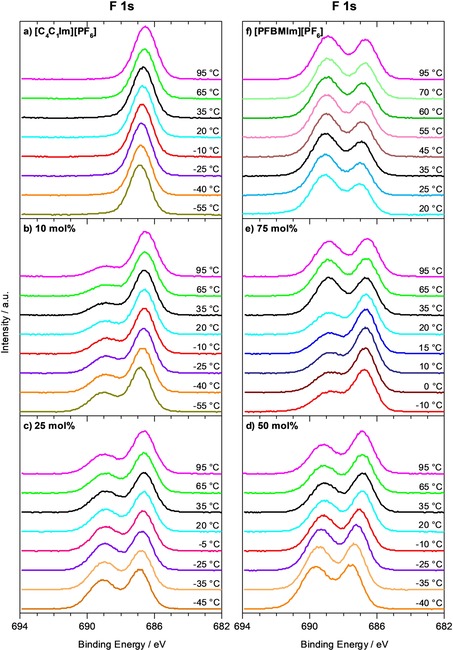
F 1s spectra measured at 80° emission, collected during cooling from 95 °C to lower temperatures: a) Neat [C_4_C_1_Im][PF_6_], b)–e) mixtures of [PFBMIm][PF_6_] with [C_4_C_1_Im][PF_6_] at molar ratios of b) 10 mol % [PFBMIm][PF_6_], c) 25 mol % [PFBMIm][PF_6_], d) 50 mol % [PFBMIm][PF_6_] and e) 75 mol % [PFBMIm][PF_6_], and f) neat [PFBMIm][PF_6_].

**Figure 5:**
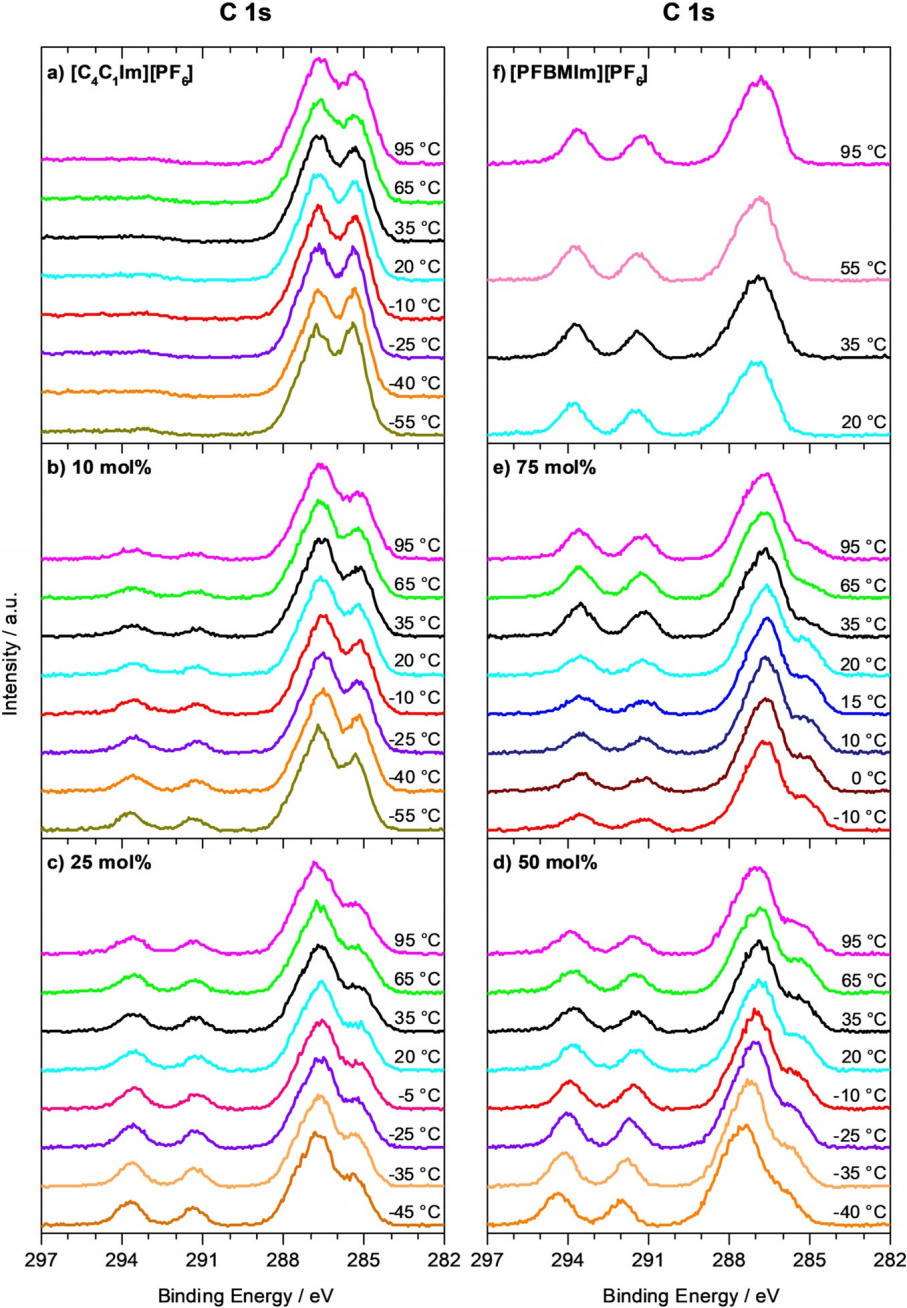
C 1s spectra measured at 80° emission, collected during cooling from 95 °C to lower temperatures: a) Neat [C_4_C_1_Im][PF_6_], b)–e) mixtures of [PFBMIm][PF_6_] with [C_4_C_1_Im][PF_6_] at molar ratios of b) 10 mol % [PFBMIm][PF_6_], c) 25 mol % [PFBMIm][PF_6_], d) 50 mol % [PFBMIm][PF_6_] and e) 75 mol % [PFBMIm][PF_6_], and f) neat [PFBMIm][PF_6_].

**Figure 6:**
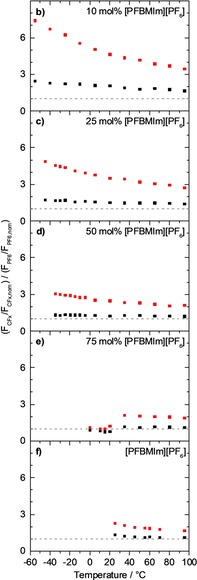
Ratio of the normalized F_CFx_ and F_PF6_ contents, at 0° (black) and 80° (red) emission angle, obtained during cooling from 95 °C to lower temperatures. a) Neat [C_4_C_1_Im][PF_6_]: Not shown, since this IL contains no F_CFx_ signal from a fluorinated chain, b)–e) mixtures of [PFBMIm][PF_6_] with [C_4_C_1_Im][PF_6_] at molar ratios of b) 10 mol % [PFBMIm][PF_6_], c) 25 mol % [PFBMIm][PF_6_], d) 50 mol % [PFBMIm][PF_6_] and e) 75 mol % [PFBMIm][PF_6_], and f) neat [PFBMIm][PF_6_]. The dashed horizontal lines indicate the nominal compositions.

As a next step, we address the temperature dependence of the XP spectra for the two neat ILs and the IL mixtures, by cooling the ILs from 95 °C down to the temperature, where solidification starts, which is typically indicated by the onset of charging. The F 1s and C 1s spectra of the neat ILs and the IL mixtures are shown in Figures 4 a–4f and 5a–5f, respectively. Due to the higher surface sensitivity, we focus on the spectra at 80°, since enrichment/depletion effects are better visible at this angle than at 0° emission. The quantitative analysis of the ratios of the normalized F_CFx_ and F_PF6_ contents at 80° and also at 0° are shown in Figure 6 b–6 f.

For neat [C_4_C_1_Im][PF_6_], we find a slight continuous decrease of the F_PF6_ signal in Figure 4 a by around 15 % upon cooling from 95 °C to −55 °C. The C_hetero’_ signal in Figure 5 a shows a comparable decrease of 8 %; in contrast to this behavior, the C_alkyl_ continuously increases. At 95 °C, the C_alkyl_ peak is smaller than the C_hetero’_ peak, but at −55 °C the intensities are reversed. This behavior indicates an increasing surface enrichment of the butyl chain with decreasing temperature. Similar effects have been observed in literature^24^ for [C_8_C_1_Im]X with X^−^=Br, [TfO], [Tf_2_N] and [C_*n*_C_1_Im][TfO] (*n=*4, 8, 18). We assign these effects to the increase in magnitude of the entropic term −*TΔS* ^*0*^ with temperature, which favors a more random distribution of the constituents with increasing temperature. This term counteracts the enthalpic driving force favoring surface enrichment of the fluorinated chain at low temperature, due to lowering of the surface tension (see above).

Upon further temperature decrease, the C_alkyl_ intensity continues to increase, until at −70 °C charging and peak broadening starts (not shown). This temperature is in accordance with the glass transition temperature of [C_4_C_1_Im][PF_6_] at around −77 °C.^20^ Subsequent heating the sample up to 95 °C yields the same spectra (within the margin of error) as observed before starting the cooling experiment.

For neat [PFBMIm][PF_6_], we again observe a slow decrease of the F_PF6_ peak at 80° during cooling from 95 to 25 °C in Figure 4 f; at the same time, the F_CFx_ intensity remains more or less unchanged. The resulting increase of the ratio of the normalized F_CFx_ and F_PF6_ contents in Figure 6 f indicates a slight increase of the surface enrichment of the fluorinated chain with decreasing temperature. No significant changes are initially observed in the C 1s region (Figure 5 f, spectra at 95 °C and 55 °C). Starting at 20 °C, peak broadening and an intensity decrease of all IL peaks indicate the onset of solidification of [PFBMIm][PF_6_]. Since bulk [PFBMIm][PF_6_] melts at 66 °C,^17^ the onset of solidification at a temperature as low as at 20 °C indicates that this IL undergoes supercooling. Upon heating the IL back to 95 °C, we observe an intensity loss of about 18 % of the F_PF6_ signal (not shown), whereas the F_CFx_ signal completely recovers (to within 3 %). A closer look to the other spectra in 80° emission shows that the N 1s XP spectrum shows a shoulder towards lower binding energy, indicating radiation damage^25^ over a prolonged exposure to X‐rays (notably, the conclusions derived here are not affected by beam damage).

Next, we discuss the behavior for the different IL mixtures upon cooling. The F 1s and C 1s spectra for selected temperatures are shown in Figures 4 b–4 e and 5 b–5e, respectively. The lowest possible temperature for XPS upon cooling depends on the molar ratio, because the solidification temperature increases with increasing [PFBMIm][PF_6_] content. Overall, the ILs with molar ratios of 10, 25 and 50 mol % [PFBMIm][PF_6_] display the same behavior. Upon cooling from 95 °C to lower temperatures, the F_CFx_ peaks gain intensity and the ratios of the normalized F_CFx_ and F_PF6_ contents for 80° in Figure 6 b–6d increase. Notably, for 0° this increase is much less pronounced (black symbols in Figure 6). The increase of the F_CFx_ intensity (see Figure 4 b–4d) detected in 80° indicates an increasing surface enrichment of the fluorinated chain of the [PFBMIm]^+^ cation in the IL mixtures upon cooling. The driving force for the increase of surface enrichment of the fluorinated chains at lower temperatures, or—in other words—the higher degree of disorder at higher temperatures in the mixtures is again attributed to the entropic contributions.

Below a certain temperature for each mixture (about −55 °C for 10 mol %, −45 °C for 25 mol % and −35 °C for 50 mol %), all signals shift towards higher binding energy and peak broadening indicates solidification and therefore charging (note that for a given mixture these temperatures were found to vary by ±5 °C due to the supercooling effects). The corresponding data is not included in Figures 4–6. The intensities of the C_CF3_ and C_CF2_ peaks in Figure 5 b–5 d increase slightly upon cooling, as expected. If the mixtures are subsequently heated to 95 °C, nearly the original F 1s and C 1s spectra are obtained, confirming reversibility of the temperature‐dependent experiment without notable changes due to prolonged X‐ray exposure.

For the molar ratio of 75 mol % [PFBMIm][PF_6_], we observe a quite different behavior. Initially, the F 1s and C 1s spectra in Figures 4 e and 5 e, respectively, and the ratio of the normalized F_CFx_ and F_PF6_ contents at 80° and also at 0° in Figure 6 e show the same continuous increase than found for the lower molar ratios. This behavior again indicates an increasing surface enrichment of the fluorinated chain of the [PFBMIm]^+^ cation in the mixture with decreasing temperature. Between 35 and 20 °C, however, the F_CFx_ intensity decreases by about 40 % while the F_PF6_ intensity stays constant. This leads to a pronounced decrease of their ratio in Figure 6 e. Upon further cooling, the ratio slightly increases again, with a similar slope than above 35 °C. Peak broadening due to charging starts at −10 °C indicating the onset of solidification of the [PFBMIm][PF_6_]‐depleted mixture. The corresponding C 1s spectra of the 75 mol % mixture (see Figure 5 e) display an intensity increase of the C_alkyl_ peak between 35 and 20 °C, whereas the C_CF2_ and C_CF3_ peaks lose intensity. No further changes are observed until the start of peak broadening at −10 °C. We assign the sharp decrease of the peaks of the fluorinated chain (F_CFx_, C_CF3_ and C_CF2_) to a partial solidification of a [PFBMIm][PF_6_]‐rich or pure [PFBMIm][PF_6_] phase, which is depleted from the outer surface beyond the XPS information depth, or even forms a solid precipitate at the buried solid/liquid interface with the support. The remaining liquid mixture is therefore [PFBMIm][PF_6_]‐depleted and [C_4_C_1_Im][PF_6_]‐rich, which leads to the decrease of the F_CFx_ signal and an increase of the C_alkyl_ signal. The peak broadening at −10 °C then indicates the solidification of this residual mixture. After subsequent heating of the mixture to 95 °C, we find the same peak intensities (within the margin of error) as before the cooling experiment; this behavior signals the re‐dissolution and mixing of [PFBMIm][PF_6_] and [C_4_C_1_Im][PF_6_] during heating the mixture. Notably, the precipitation of one of the ILs from the mixture was only observed for high [PFBMIm][PF_6_] content such as 75 mol % (a very similar behavior was also detected for a 85 mol % mixture, see Figure S2 in the Supporting Information), and not in any of the other mixtures (10, 25 and 50 mol % mixtures). It should be noted that the magnitude of the abrupt decrease of the ratio in Figure 6 e between 35 and 20 °C varied from experiment to experiment. Such a behavior is typical for liquids that show supercooling, as slight contaminations or other disturbances might induce or prevent spontaneous solidification. Recently, a similar phenomenon was reported for mixtures of Cs[Tf_2_N] and [PPh_4_][Tf_2_N], where the temperature‐dependent depletion of tetraphenylphosphonium, [PPh_4_]^+^, from the near‐surface region (information depth) was observed by XPS.^19^ In order to correlate surface phenomena as described in this last section with liquid‐solid phase transitions in the bulk of IL mixtures in more detail, a combination of surface‐sensitive ARXPS with other methods such as differential scanning calorimetry, scattering or microscopy techniques would be very helpful, but are out of the scope of this article.

## Conclusions

We used angle‐resolved X‐ray photoelectron spectroscopy to study mixtures of fluorinated and non‐fluorinated ILs, namely [PFBMIm][PF_6_] and [C_4_C_1_Im][PF_6_], with molar ratios of 10, 25, 50 and 75 mol % [PFBMIm][PF_6_], along with the two neat ILs, at 95 °C and while cooling to lower temperatures. The two ILs contain the same anion, [PF_6_]^−^, but different cations. By performing very surface‐sensitive measurements at an emission angle of 80°, we observe surface enrichment of the fluorinated chain for neat [PFBMIm][PF_6_] relative to the bulk composition. This effect is in line with the general observation that fluorinated groups in the outermost layer lead to a lower surface tension than alkyl groups.1b, 6a, 23 The lower the molar ratio of [PFBMIm][PF_6_] in the mixture, the more pronounced is the surface enrichment of the fluorinated chain relative to the bulk composition. Upon cooling the mixtures from their liquid phase at 95 °C to their solidification, we observe a similar behavior for the 10, 25 and 50 mol % mixtures, and also for neat [PFBMIm][PF_6_]: Decreasing the temperature leads to an increase in surface enrichment of the fluorinated chain of [PFBMIm][PF_6_] (relative to the bulk composition), which is detected in the F 1s and C 1s spectra. We attribute the observed behavior to entropic reasons, namely a less pronounced enrichment of the fluorinated chains, that is, a lower degree of order, at high temperatures. The 75 mol % mixture shows the same increase in enrichment of the fluorinated chain as the other mixtures when cooling down to 35 °C. Upon further cooling to 20 °C, however, the surface enrichment of the fluorinated chain decreases drastically. This observation is attributed to a (partial) precipitation of the pure [PFBMIm][PF_6_], which in turn results in a [C_4_C_1_Im][PF_6_]‐rich phase at the topmost layer of the mixture. This change in composition is also reflected by an increase of the C_alkyl_ peak in the C 1s spectrum when comparing the 35 and 20 °C spectra.

## Conflict of interest

The authors declare no conflict of interest.

## Supporting information

As a service to our authors and readers, this journal provides supporting information supplied by the authors. Such materials are peer reviewed and may be re‐organized for online delivery, but are not copy‐edited or typeset. Technical support issues arising from supporting information (other than missing files) should be addressed to the authors.

Supplementary

## References

[1a] A. B. Pereiro , M. J. Pastoriza-Gallego , K. Shimizu , I. M. Marrucho , J. N. Canongia Lopes , M. M. Piñeiro , L. P. N. Rebelo , J. Phys. Chem. B 2013, 117, 10826–10833; 23964834 10.1021/jp402300c

[1b] A. Luís , K. Shimizu , J. M. M. Araújo , P. J. Carvalho , J. A. Lopes-da-Silva , J. N. Canongia Lopes , L. P. N. Rebelo , J. A. P. Coutinho , M. G. Freire , A. B. Pereiro , Langmuir 2016, 32, 6130–6139; 27218210 10.1021/acs.langmuir.6b00209 PMC5325320

[1c] T. L. Greaves , D. F. Kennedy , Y. Shen , A. Hawley , G. Song , C. J. Drummond , Phys. Chem. Chem. Phys. 2013, 15, 7592–7598; 23588776 10.1039/c3cp44589e

[1d] L. Zhou , J. Fan , X. Shang , Materials 2014, 7, 3867–3880. 28788654 10.3390/ma7053867 PMC5453234

[2] M. L. Ferreira , M. J. Pastoriza-Gallego , J. M. M. Araújo , J. N. Canongia Lopes , L. P. N. Rebelo , M. M. Piñeiro , K. Shimizu , A. B. Pereiro , J. Phys. Chem. C 2017, 121, 5415–5427.

[3] S. Tsuzuki , T. Umecky , H. Matsumoto , W. Shinoda , M. Mikami , J. Phys. Chem. B 2010, 114, 11390–11396. 20707368 10.1021/jp104380s

[4] J. van den Broeke , F. Winter , B.-J. Deelman , G. van Koten , Org. Lett. 2002, 4, 3851–3854. 12599475 10.1021/ol026700l

[5] O. Hollóczki , M. Macchiagodena , H. Weber , M. Thomas , M. Brehm , A. Stark , O. Russina , A. Triolo , B. Kirchner , ChemPhysChem 2015, 16, 3325–3333. 26305804 10.1002/cphc.201500473 PMC4641458

[6a] E. J. Smoll, Jr. , M. A. Tesa-Serrate , S. M. Purcell , L. D′Andrea , D. W. Bruce , J. M. Slattery , M. L. Costen , T. K. Minton , K. G. McKendrick , Faraday Discuss. 2018, 206, 497–522; 28944811 10.1039/c7fd00175d

[6b] Y. Zhang , Y. Khalifa , E. J. Maginn , J. T. Newberg , J. Phys. Chem. C 2018, 122, 27392–27401;

[6c] D. W. Bruce , C. P. Cabry , J. N. C. Lopes , M. L. Costen , L. D′Andrea , I. Grillo , B. C. Marshall , K. G. McKendrick , T. K. Minton , S. M. Purcell , S. Rogers , J. M. Slattery , K. Shimizu , E. Smoll , M. A. Tesa-Serrate , J. Phys. Chem. B 2017, 121, 6002–6020. 28459567 10.1021/acs.jpcb.7b01654

[7] ECHA—European Chemicals Agency, echa.europa.eu, accessed June 2019; personal communication with proionic GmbH.

[8a] M. T. Clough , C. R. Crick , J. Gräsvik , P. A. Hunt , H. Niedermeyer , T. Welton , O. P. Whitaker , Chem. Sci. 2015, 6, 1101–1114; 29560198 10.1039/c4sc02931c PMC5811077

[8b] H. Niedermeyer , J. P. Hallett , I. J. Villar-Garcia , P. A. Hunt , T. Welton , Chem. Soc. Rev. 2012, 41, 7780–7802. 22890419 10.1039/c2cs35177c

[9a] M. Chakraborty , T. Ahmed , R. S. Dhale , D. Majhi , M. Sarkar , J. Phys. Chem. B 2018, 122, 12114–12130; 30495959 10.1021/acs.jpcb.8b09699

[9b] R. S. Dhale , P. K. Sahu , M. Sarkar , J. Phys. Chem. B 2017, 121, 7934–7945; 28715188 10.1021/acs.jpcb.7b04585

[9c] D. C. Khara , J. P. Kumar , N. Mondal , A. Samanta , J. Phys. Chem. B 2013, 117, 5156–5164; 23544669 10.1021/jp400914y

[9d] S. N. Baker , G. A. Baker , F. V. Bright , Green Chem. 2002, 4, 165–169;

[9e] S. K. Das , P. K. Sahu , M. Sarkar , J. Fluoresc. 2013, 23, 1217–1227. 23813188 10.1007/s10895-013-1252-4

[10a] B. S. J. Heller , C. Kolbeck , I. Niedermaier , S. Dommer , J. Schatz , P. Hunt , F. Maier , H.-P. Steinrück , ChemPhysChem 2018, 19, 1733–1745; 29645340 10.1002/cphc.201800216 PMC6175172

[10b] M. B. Oliveira , M. Domínguez-Pérez , O. Cabeza , J. A. Lopes-da-Silva , M. G. Freire , J. A. P. Coutinho , J. Chem. Thermodyn. 2013, 64, 22–27; 10.1021/jp3059905 22963646

[10c] C. Ridings , G. G. Warr , G. G. Andersson , J. Phys. Chem. Lett. 2017, 8, 4264–4267. 28823156 10.1021/acs.jpclett.7b01654

[11] F. Wu , W. V. Karunaratne , C. J. Margulis , J. Phys. Chem. C 2019, 123, 4914–4925.

[12a] K. Nakajima , M. Miyashita , M. Suzuki , K. Kimura , J. Chem. Phys. 2013, 139, 224701; 24329076 10.1063/1.4838376

[12b] R. Souda , Surf. Sci. 2010, 604, 1694–1697.

[13a] K. Nakajima , S. Oshima , M. Suzuki , K. Kimura , Surf. Sci. 2012, 606, 1693–1699;

[13b] K. Nakajima , S. Nakanishi , Z. Chval , M. Lísal , K. Kimura , J. Chem. Phys. 2016, 145, 184704; 27846689 10.1063/1.4967260

[13c] K. Nakajima , S. Nakanishi , M. Lísal , K. Kimura , J. Mol. Liq. 2017, 230, 542–549.

[14] I. J. Villar-Garcia , S. Fearn , N. L. Ismail , A. J. S. McIntosh , K. R. J. Lovelock , Chem. Commun. 2015, 51, 5367–5370. 10.1039/c4cc06307d 25236677

[15a] I. J. Villar-Garcia , K. R. J. Lovelock , S. Men , P. Licence , Chem. Sci. 2014, 5, 2573–2579;

[15b] S. Men , P. Licence , Chem. Phys. Lett. 2017, 681, 40–43;

[15c] F. Maier , T. Cremer , C. Kolbeck , K. R. J. Lovelock , N. Paape , P. S. Schulz , P. Wasserscheid , H.-P. Steinrück , Phys. Chem. Chem. Phys. 2010, 12, 1905–1915; 20145858 10.1039/b920804f

[15d] S. Men , P. Licence , Chem. Phys. Lett. 2017, 686, 74–77;

[15e] S. Men , K. R. J. Lovelock , P. Licence , Phys. Chem. Chem. Phys. 2011, 13, 15244–15255. 21779587 10.1039/c1cp21053j

[16a] S. Palchowdhury , B. L. Bhargava , Phys. Chem. Chem. Phys. 2015, 17, 19919–19928; 26166036 10.1039/c5cp02932e

[16b] S. Palchowdhury , B. L. Bhargava , J. Phys. Chem. B 2016, 120, 5430–5441.

[17] M. Lexow , B. S. J. Heller , G. Partl , R. G. Bhuin , F. Maier , H.-P. Steinrück , Langmuir 2019, 35, 398–405. 30540199 10.1021/acs.langmuir.8b03517 PMC6377181

[18] I. Niedermaier , C. Kolbeck , H.-P. Steinrück , F. Maier , Rev. Sci. Instrum. 2016, 87, 045105. 27131705 10.1063/1.4942943

[19] R. G. Bhuin , P. Schreiber , B. S. J. Heller , M. Scheuermeyer , P. Wasserscheid , H.-P. Steinrück , F. Maier , J. Mol. Liq. 2019, 275, 290–296.

[20] P. B. P. Serra , F. M. S. Ribeiro , M. A. A. Rocha , M. Fulem , K. Růžička , J. A. P. Coutinho , L. M. N. B. F. Santos , J. Mol. Liq. 2017, 248, 678–687.

[21a] C. Kolbeck , M. Killian , F. Maier , N. Paape , P. Wasserscheid , H.-P. Steinrück , Langmuir 2008, 24, 9500–9507; 18672915 10.1021/la801261h

[21b] J. M. Gottfried , F. Maier , J. Rossa , D. Gerhard , P. S. Schulz , P. Wasserscheid , H.-P. Steinrück , Z. Phys. Chem. 2006, 220, 1439–1453. 10.1002/anie.200602756 17061300

[22a] C. Kolbeck , I. Niedermaier , A. Deyko , K. R. J. Lovelock , N. Taccardi , W. Wei , P. Wasserscheid , F. Maier , H.-P. Steinrück , Chem. Eur. J. 2014, 20, 3954–3965; 24643947 10.1002/chem.201304549

[22b] K. R. J. Lovelock , C. Kolbeck , T. Cremer , N. Paape , P. S. Schulz , P. Wasserscheid , F. Maier , H.-P. Steinrück , J. Phys. Chem. B 2009, 113, 2854–2864. 19708216 10.1021/jp810637d

[23] T. L. Merrigan , E. D. Bates , S. C. Dorman , J. H. Davis Jr , Chem. Commun. 2000, 2051–2052.

[24] C. Kolbeck , A. Deyko , T. Matsuda , F. T. U. Kohler , P. Wasserscheid , F. Maier , H.-P. Steinrück , ChemPhysChem 2013, 14, 3726–3730. 24123477 10.1002/cphc.201300719

[25a] T. Cremer , M. Stark , A. Deyko , H.-P. Steinrück , F. Maier , Langmuir 2011, 27, 3662–3671; 21361299 10.1021/la105007c

[25b] C. Kolbeck , T. Cremer , K. R. J. Lovelock , N. Paape , P. S. Schulz , P. Wasserscheid , F. Maier , H.-P. Steinrück , J. Phys. Chem. B 2009, 113, 8682–8688. 19534566 10.1021/jp902978r

