# Supplementary material for: Temperature‐Dependent Surface Enrichment Effects in Binary Mixtures of Fluorinated and Non‐Fluorinated Ionic Liquids
Source: Chemistry. 2020 Jan 7;26(5):1117–26. doi: 10.1002/chem.201904438 (PMC7027538; doi:10.1002/chem.201904438)
Supplement: Supplementary file 1 — Supplementary [file CHEM-26-1117-s001.pdf]

CHEMISTRY
A European Journal

Supporting Information

Temperature-Dependent Surface Enrichment Effects in Binary Mixtures of Fluorinated and Non-Fluorinated Ionic Liquids

Bettina S. J. Heller,[a] Matthias Lexow,[a] Francesco Greco,[a] Sunghwan Shin,[a] Gabriel Partl,[b] Florian Maier,[a] and Hans-Peter Steinrück*[a]

chem_201904438_sm_miscellaneous_information.pdf

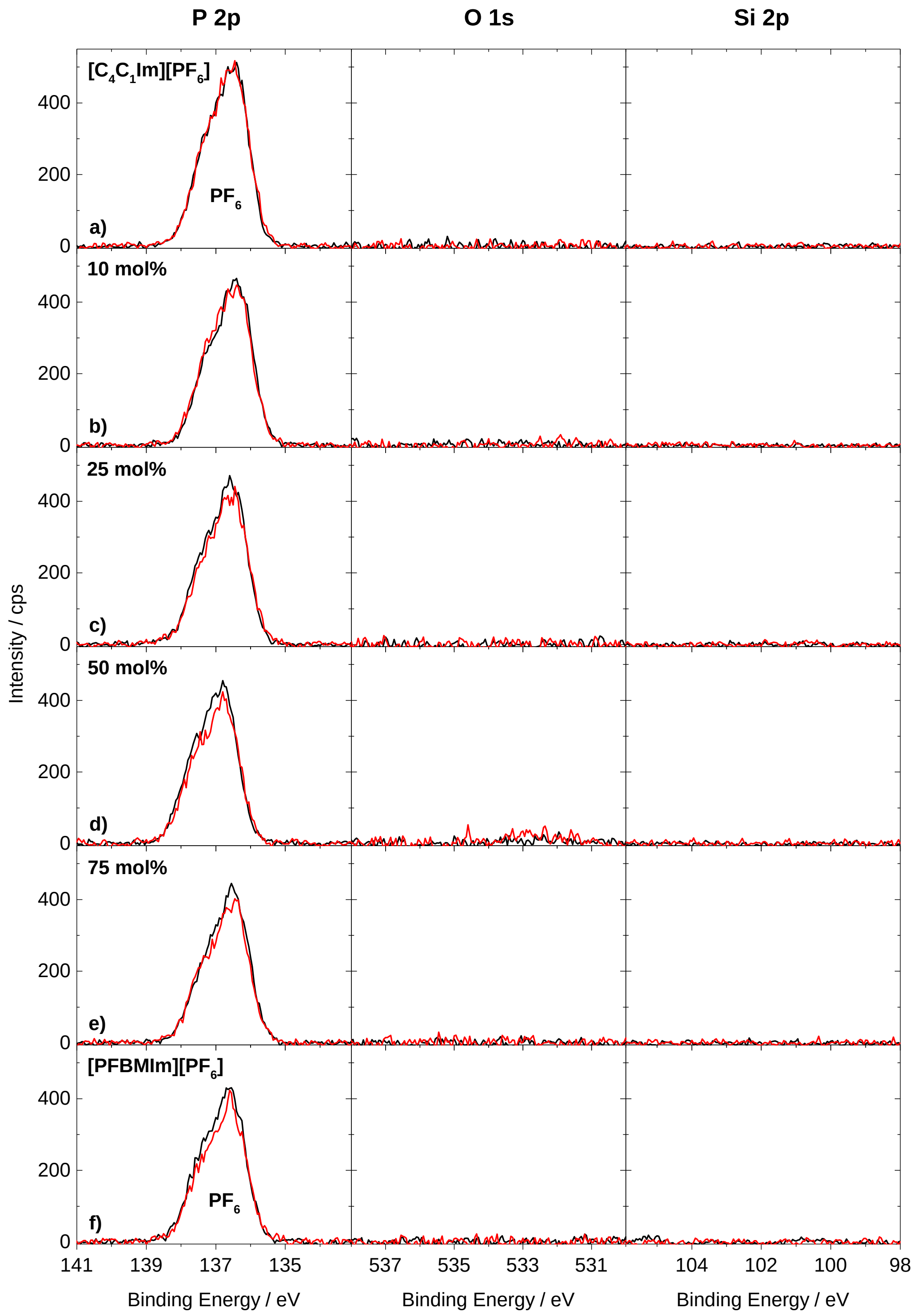

Figure S1: P 2p (left), O 1s (center) and Si 2p (right) spectra, at 0° (black) and 80° (red) emission: a) Neat $[C_4C_1Im][PF_6]$, b) – e) mixtures of $[C_4C_1Im][PF_6]$ with $[PFBMIm][PF_6]$ at molar ratios of b) 10 mol% $[PFBMIm][PF_6]$, c) 25 mol% $[PFBMIm][PF_6]$, d) 50 mol% $[PFBMIm][PF_6]$ and e) 75 mol% $[PFBMIm][PF_6]$, and f) neat $[PFBMIm][PF_6]$. In all cases, the sample temperature was 95 °C.

Table S1: List of the eight 1-ethyl-3-methylimidazolium $[C_2C_1Im][X]$ ILs, registered by REACH in June 2019.

[X] = [benzoate]$^-$, registered by BASF SE

= $[MeSO_3]^-$, methanesulfonate, registered by BASF SE

= $[EtSO_4]^-$, ethylsulphate, registered by BASF SE

= [Otf]$^-$, trifluoromethanesulfonate, registered by BASF SE

= Cl^-, chloride registered by BASF SE

= [DCA]$^-$, N-cyanocyanamide, registered by BASF SE

= $[BF_4]^-$, tetrafluoroborate, registered by proionic GmbH

= [OAc]$^-$, acetate, registered by proionic GmbH

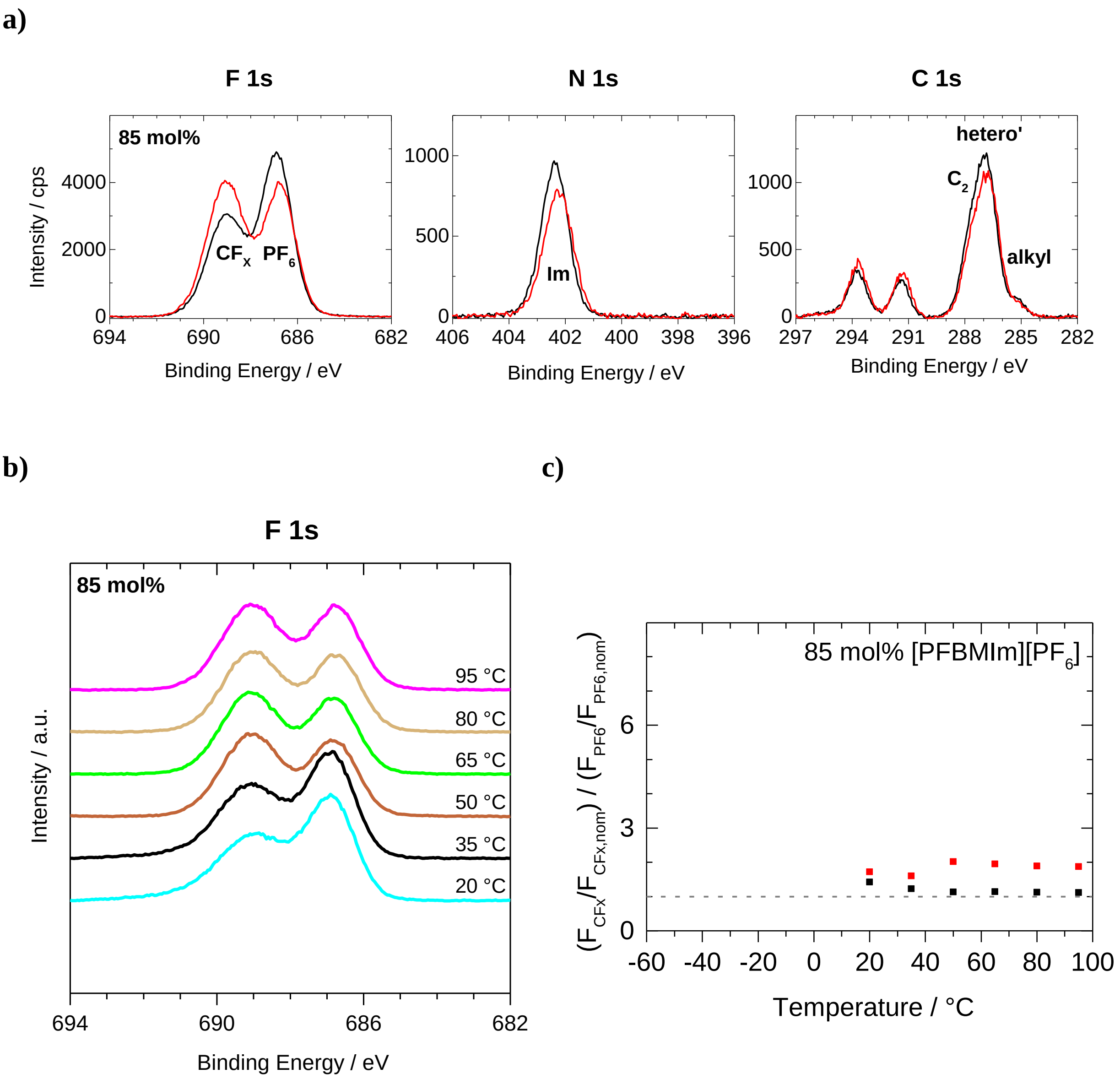

Figure S2: Mixture of [PFBMIm][PF_6] and [C_4C_1Im][PF_6] at a molar ratio of 85 mol% [PFBMIm][PF_6]. a) F 1s (left), N 1s (center) and C 1s (right) spectra, at 0° (black) and 80° (red) emission, acquired at a sample temperature of 95 °C. b) F 1s spectra measured at 80° emission, collected during cooling from 95 °C to lower temperatures. c) Ratio of the normalized F_{CFx} and F_{PF6} contents, obtained from spectra at 0° (black) and 80° (red) emission angle during cooling from 95 °C to lower temperatures.
